# Rompendo o silêncio branco: não basta ser antirracista, é preciso agir

**DOI:** 10.1590/0102-311XPT137224

**Published:** 2024-11-04

**Authors:** Carolina Magalhães Heringer, Tatiana Wargas de Faria Baptista

**Affiliations:** 1 Instituto Nacional de Saúde da Mulher, da Criança e do Adolescente Fernandes Figueira, Fundação Oswaldo Cruz, Rio de Janeiro, Brasil.; 2 Escola Nacional de Saúde Pública Sergio Arouca, Fundação Oswaldo Cruz, Rio de Janeiro, Brasil.

Nosso encontro com o livro ^1^ surgiu da nossa inquietação em relação ao lugar de privilégio branco e sua perpetuação social e estrutural. Esse foi um encontro entre duas mulheres brancas - que estão em busca de reconhecer e romper com seus privilégios - com uma variedade de debates que geram desconfortos necessários. Também acreditamos que outra sociedade é possível e urgente, de forma que devemos assumir a responsabilidade de agir diante das dimensões estruturais, institucionais e individuais do racismo, pois “*o racismo não é uma problemática pessoal, mas um problema branco estrutural e institucional que pessoas negras experienciam*” ^2^ (p. 204).

Os diálogos apresentados no livro, organizado por Lia Vainer Schucman, originam-se de encontros realizados nos anos de 2020 e 2021, promovidos pelo Instituto Ibirapitanga ^3^, entre pensadores e ativistas que se dedicam à luta antirracista e expressam desconfortos diante da perpetuação da branquitude. As conversas aconteceram em seis momentos e, em cada um deles, havia dois ou três responsáveis pelo debate. São conversas que nos provocam e conclamam a pensar sobre como corpos brancos mantêm seus privilégios, supostas superioridades e violências, eximindo-se da responsabilidade com o que provocam de sofrimento e morte. Em cada texto/debate, o pacto da branquitude ^4^ e sua contraface, o racismo, são expostos em minúcias, com o objetivo de tecer estratégias para avançar na luta antirracista e na quebra desse pacto perverso que continua velado para muitos.

Todos os diálogos entrelaçam-se, complementam-se, sustentam-se e reafirmam-se em confiança e aliança, construída por passos anteriores que abriram caminhos para a sobrevivência e a resistência da população negra. Aliança entre os negros que vêm de longe, lutaram, e seguem lutando contra a violência e os privilégios brancos. Como convocam Winnie Bueno, Ronilso Pacheco & Lia Vainer Schucman, é preciso entender que a diáspora é um sentimento que faz parte da luta por direitos e das profundas reivindicações que duram anos.

O debate entre Sueli Carneiro & Lia Schucman, mediado por Ana Paula Lisboa, desperta a reflexão sobre as alianças de dor e resistência, mas também as alianças de violência e extermínio. Visto que, desde a colonização, os brancos se associaram para explorar, violentar e expropriar a população negra, tirando vantagens sobre ela. Ambas as alianças se reconfiguram e se mantêm até os dias de hoje, sendo necessário questionar: em qual aliança estamos? Quais as vantagens que foram construídas pelos brancos a partir do pacto da violência? E quais os rebatimentos dessas alianças sobre a saúde da população negra?

Em vários momentos, os autores ressaltam que a saúde da população negra é prejudicada cotidianamente pelo racismo e pelo pacto da branquitude. As pessoas negras enfrentam dificuldades no acesso às instituições de saúde, com atendimentos negados, negligenciados e/ou atravessados por violências racistas. As instituições têm o seu funcionamento baseado em vantagens e desvantagens conforme a raça ^4^ ^,^ ^5^. Portanto, o racismo e a branquitude podem determinar o adoecimento e a morte das pessoas negras.

Na conversa protagonizada por Robin DiAngelo, Cida Bento & Thiago Amparo, podemos refletir sobre as vidas negras como sobreviventes de um pacto de morte construído pelos brancos para se manter no poder acima de tudo e de todos os não brancos. Deivison Faustino, Lourenço Cardoso & Luciana Brito mobilizam Frantz Fanon para explicitar o processo de racialização construído pelos brancos, que impõe aos negros lugares de inferiorização, marginalização e desumanização, numa “zona de não ser” ^6^ que impacta toda a sua vida e saúde. Faustino et al. também problematizam que a ideia de “universal” é racializada e que isso tem reflexos na área da saúde. Os autores compartilham o seguinte: “*se o movimento negro não brigar muito dentro do espaço da saúde, a ideia de universalidade em si acaba refletindo exclusivamente a experiência dos brancos, mesmo que seja dos brancos pobres* (...). *As mulheres pretas morrem mais que as brancas em todos os estados do Brasil. Se uma política de combate à morte materna não tem dimensão racial em vista, ela acaba beneficiando apenas as mulheres brancas, porque as nossas desigualdades sociais criam essa estrutura e fazem com que o branco seja privilegiado nesse espaço*” (p. 101-2).

Esse destaque já diz tudo. E, em concordância, afirmamos que, na intersecção do racismo e do sexismo institucional, as mulheres negras têm grandes prejuízos à saúde. Uma das expressões desses prejuízos são as mortes maternas. O óbito materno é em torno de 90% evitável, sendo uma violação dos direitos humanos das mulheres. As mulheres negras são as maiores vítimas de mortes maternas no Brasil, apresentando um risco de morte duas vezes maior que o das mulheres brancas ^7^. Com isso, esses índices nos fazem questionar o acesso universal à saúde e aos direitos humanos.

Refletindo sobre as problemáticas do racismo na saúde, a partir dos diálogos expostos no livro, revisitamos o texto *Racismo Institucional e Saúde da População Negra* de Jurema Werneck ^8^ e reconhecemos que, embora as reivindicações da população negra na luta por um sistema universal de saúde tenham sido fundamentais, elas não garantiram o acesso equitativo à saúde. Ainda hoje, a luta por equidade é necessária, visto que a população negra representa a maioria dos usuários do Sistema Único de Saúde (SUS). A equidade em saúde só é possível com uma compreensão crítica sobre o protagonismo violento da branquitude, que é mantido historicamente por meio do racismo.

Bueno et al. evidencia que vivemos em uma sociedade sustentada pelo terror racial e caracterizada pelo genocídio antinegro. Para romper com essa lógica desumana, é necessária uma rebelião advinda da união do movimento negro e das pessoas antirracistas. Nesse sentido, o combate ao racismo deve ser uma responsabilidade dos brancos. Como destaca Djamila Ribeiro ^9^ (p. 39), a partir de Audre Lorde, “*é necessário matar o opressor que há em nós, e isso não é feito apenas se dizendo antirracista: é preciso fazer cobranças*”. Precisamos estar dispostos a renunciar aos nossos privilégios brancos e agir na luta antirracista.

Não podemos mais compactuar com essa manutenção de violências e iniquidades. Não podemos mais ser cúmplices de tanto sangue derramado, tantas mortes e tantos adoecimentos cotidianos. Devemos ir à raiz do problema, que é o branco e sua branquitude. Precisamos agir para ontem, para hoje e para amanhã, de forma coletiva, envolvente e reparadora.

Os debates apresentados no livro são provocativos à mudança e à responsabilização dos brancos. Precisamos assumir nossas responsabilidades, agir e romper com o pacto da branquitude. Concordando com Jurema Werneck, Thula Pires & Bianca Santana, é essencial e urgente romper com esse mundo e construir um novo. E, por favor, não leiam isso de forma ilusória ou utópica: é possível, necessário e urgente. Novos caminhos são factíveis e inadiáveis.

## References

[1] Schucman LV, Instituto Ibirapitanga (2023). Branquitude: diálogos sobre racismo e antirracismo.

[2] Kilomba G (2019). Memórias da plantação. Episódios de racismo cotidiano.

[3] Instituto Ibirapitanga Encontro Branquitude: Racismo e Antirracismo.. Vídeo.

[4] Bento C (2022). O pacto da branquitude.

[5] Almeida S (2019). Racismo estrutural.

[6] Fanon F (2022). Os condenados da terra.

[7] Leal MC, Granado S, Bittencourt S, Esteves AP, Caetano K (2023). Nascer no Brasil II: pesquisa nacional sobre aborto, parto e nascimento
2022-2023.

[8] Werneck J (2016). Racismo institucional e saúde da população negra. Saúde Soc.

[9] Ribeiro D (2019). Pequeno manual antirracista.

